# MRPL18 Promotes Breast Cancer Progression: Connecting Mitochondrial Ribosomal Protein to Immune Response

**DOI:** 10.32604/or.2025.065050

**Published:** 2025-08-28

**Authors:** Hailong Li, Wen Ouyang, Yiyin Long, Yun Peng, Ziyi Liu, Qi Zhou, Rong Xu, Wei Du

**Affiliations:** 1 Department of Pathology, Changde Hospital, Xiangya School of Medicine, Central South University (The First People’s Hospital of Changde City), Changde, 415000, China; 2 Medical Department, Changde Hospital, Xiangya School of Medicine, Central South University (The First People’s Hospital of Changde City), Changde, 415000, China

**Keywords:** Breast cancer, mitochondrial ribosomal protein L18 (MRPL18), prognostic biomarker, therapeutic target, immune infiltration

## Abstract

**Background:**

The study aimed to explore the clinical value of mitochondrial ribosomal protein L18 (MRPL18) in breast cancer.

**Methods:**

Multiple databases were used to validate the expression of MRPL18. The prognostic impact and predictive value of MRPL18 were evaluated by using predictive models. Protein-protein interaction (PPI) networks were constructed by using GeneMANIA. Enrichment analysis is used to explore the signaling pathway regulated by MRPL18. Cell counting kit-8 (CCK-8) assays, colony formation, migration assays, flow cytometry, and xenograft models were employed to evaluate the role of MRPL18 in tumor progression. The immune response of MRPL18 was examined using correlation analysis.

**Results:**

High levels of MRPL18 are considered a risk factor in breast cancer. *In vitro* and *in vivo* studies demonstrated that MRPL18 promotes proliferation and migration in breast cancer. Besides, results found that MRPL18 promotes tumor growth through the activation of phosphatidylinositol 3-kinase (PI3K). Furthermore, high MRPL18 expression was linked to reduced immunotherapy efficacy, as indicated by correlations with immune checkpoints and immune-infiltrating patterns.

**Conclusion:**

MRPL18 promotes the progression of breast cancer.

## 1 Introduction

Breast cancer is now the most prevalent cancer among women worldwide, surpassing lung cancer [1]. Despite advances in treatment, many patients still experience poor outcomes due to issues such as recurrence and metastasis [2]. The emergence of precision medicine has underscored the importance of personalized treatment strategies and comprehensive patient assessments [3]. Nonetheless, the prognosis and treatment options for breast cancer are primarily determined by TNM staging and molecular subtyping [4]. The complexity and variability of breast cancer make it vital to identify new biomarkers and therapeutic targets to better predict prognosis and inform treatment strategies.

Mitochondrial ribosomal protein L18 (MRPL18), also referred to as L18mt, uL18m, HSPC071, and MRP-L18, is located on chromosome 6q25.3 and encodes a component of the 39S subunit of the mitochondrial ribosome [5]. MRPL18 expression was improperly regulated, resulting in lower survival and tumor purity in non-small cell lung cancer, whereas in healthy older adults, the gene’s expression in tissues went the opposite way [6]. Besides, MRPL18 plays a role in colorectal mucosal carcinoma, with elevated levels observed in normal colon epithelial cells under high glucose conditions [7]. MRPL18’s involvement in the cytosolic stress response in mammalian cells, a key mechanism for cancer survival and progression [8]. Nonetheless, the involvement of MRPL18 in cancer development and its molecular pathways are still not well understood.

This study examined MRPL18’s role in breast cancer via numerous experiments and explored the expression level of MRPL18 and its clinical value using bioinformatics. This study’s findings could aid in assessing MRPL18’s potential as a therapeutic target for breast cancer.

## 2 Materials and Methods

### 2.1 Data Acquisition

The Cancer Genome Atlas provided the breast cancer patient data (TCGA, https://portal.gdc.cancer.gov/) and Genotype-Tissue Expression (GTEx) (https://www.gtexportal.org/home/) database [9]. GSE109169 datasets were downloaded from the Gene Expression Omnibus (GEO, https://www.ncbi.nlm.nih.gov/geo/) database and the Human Protein Atlas (HPA, http://www.proteinatlas.org/) database was used for validation [10]. Data were preprocessed and log-transformed using R packages. The MRPL18 RNA expression was investigated using the ConsensusPathDB (https://cpdb.molgen.mpg.de/) and GTEx datasets, while the FANTOM5 dataset (https://fantom.gsc.riken.jp/) was employed to analyze MRPL18 protein expression levels. 60 pairs of breast tissues, comprising normal and cancerous samples, were gathered from The First People’s Hospital of Changde City (Changde, China). The Ethics Committees of The First People’s Hospital of Changde City approved all sample collection procedures (ethics approval: 2024-147-01).

### 2.2 Analysis of Single-Cell Expression and Immune Cell Type Specificity

The analysis of all protein-coding genes was conducted using single-cell RNA sequencing (scRNA-seq), resulting in their classification into 15 distinct cell groups (https://www.proteinatlas.org/MRPL18/single+cell+type (accessed on 02 June 2025)) [11].

Analyze the specificity of different types of immune cells by referring to the immune cell section in the Human Protein Atlas (https://www.proteinatlas.org/immune+cell (accessed on 02 June 2025)). This study examined various immune cell lineages and total peripheral blood mononuclear cells (PBMCs). The datasets from Monaco and Schmiedel were used to analyze MRPL18 expression across different blood cell lineages [12].

### 2.3 Immunohistochemical (IHC) Analysis

Using a microtome (Leica RM2235), the paraffin blocks were sliced into Section 3 μm thick. To remove paraffin, the sections were subjected to a series of xylene washes (2 × 10 min) followed by rehydration in graded ethanol (100%, 90%, 70%; 2 × 5 min each) and finally rinsed in distilled water. Antigen retrieval was conducted. Sections were then incubated for an hour at room temperature using a blocking buffer that included 5% bovine serum albumin to decrease nonspecific binding. Afterward, the sections were incubated using the MRPL18 primary antibody (Atlas Antibodies, HPA028775, 1:200, Solna, Sweden) overnight. The sections were washed 5 times with PBS (pH 7.3, ×1) after incubation with secondary antibodies (Thermo Fisher Scientific, A11034, 1:1000, Kitchener, ON, Canada). Positive staining was quantified in five randomly selected microscopic fields. The immunohistochemistry results were evaluated by at least three experienced pathologists [13].

### 2.4 Clinicopathological Characteristics Analysis

Using logistic regression analysis, the connection between MRPL18 expression levels and clinicopathological features was evaluated. To determine the diagnostic relevance of MRPL18, the ROC curve was employed with the help of the ‘pROC’ package (v1.18.0) and visualized through ‘ggplot2’ (v3.4.2). For Kaplan-Meier survival analyses, the ‘survival’ (v3.5–5) R packages were employed, using the ‘surv_cutpoint’ function to establish cutoffs.

### 2.5 Cox Risk Regression Analyses

To identify independent predictors of overall survival (OS) in patients, both univariate and multivariate Cox regression analyses were carried out. The analyzed variables included histological type, T, N, and M stages, PR and ER statuses, PAM50 subtype, menopause status, anatomic neoplasm subdivisions, and MRPL18 expression.

### 2.6 Protein-Protein Interaction (PPI) and Functional Enrichment Analysis

The GeneMANIA website (https://genemania.org/) was utilized for analyzing the PPI network [14]. The ‘clusterProfiler’ package (v4.4.4) was utilized for Kyoto Encyclopedia of Genes and Genomes (KEGG) and Gene Ontology (GO) analyses, as previously described [15].

### 2.7 Cell Lines and Their Respective Culture Conditions

The MDA-MB-231 (HTB-26) and BT-549 (HTB-122) cell lines, known for their high invasiveness as triple-negative breast cancer models, were sourced from the specialized company (ATCC, Manassas, VA, USA). Additionally, routine checks for Mycoplasma contamination and STR identification were performed before starting any experimental activities. All cell lines were cultured in a humidified incubator by Thermo Fisher Scientific at 37°C with 5% CO_2_.

### 2.8 Cell Transfection

RiboBio Co., Ltd. (Guangzhou, China) designed and synthesized the siRNA specifically aimed at MRPL18. Qingke Biotechnology Co., Ltd. (Beijing, China) developed the MRPL18 overexpression vector by incorporating the complete MRPL18 coding sequence into the pcDNA3.1 plasmid. Lipofectamine™ 2000 (11668030, Thermo Fisher Scientific, Waltham, MA, USA) was used for transfection with either 50 nm siRNA or a 2 μg MRPL18 overexpression vector. The culture medium was refreshed eight hours post-transfection. The primer sequences were synthesized by Qingke Biotechnology Co., Ltd. (Beijing, China) and were listed in the Table S1.

### 2.9 Detection of Transfection Efficiency and Signaling Pathway via Western Blotting Assay

The protein sample, weighing 35 μg, was processed through SDS-PAGE and subsequently transferred to a Polyvinylidene fluoride (PVDF) membrane (MilliporeSigma, 05317, Louis, MO, USA). The membrane was followed by an overnight incubation with primary antibodies: MRPL18 (1:500, ZEN BIO, Chengdu, China), Phospho-PI3 Kinase (1:1000, CST, Boston, MA, USA, 4228), Phospho-AKT (1:1000, CST, MA, USA, 9271), and GAPDH (1:1000, Proteintech, Wuhan, China). The membrane was incubated with HRP-conjugated goat anti-rabbit IgG secondary antibody (ZEN BIO, Chengdu, China; 1:3000) for 45 min. The protein bands were visualized using the Tanon 5200 chemiluminescence imaging system (Tanon Science and Technology Co., Ltd., Shanghai, China) after adding the chemiluminescent substrate (Biosharp, BL520A, Hefei, China). Protein expression levels were determined by quantifying band intensities with the help of ImageJ software (version 1.52a, NIH, Bethesda, MD, USA).

### 2.10 Cell Proliferation and Migration Assays

Cell Counting Kit-8 (CCK-8) assay: A total of 1000 cells (MDA-MB-231 and BT-549) were seeded into each well of a 96-well plate. After cell attachment, 10 μL of CCK-8 reagent (CA1210-100T, Solarbio, Beijing, China) was added to each well. Colony formation assay: The experiment involved seeding 1000 cells (MDA-MB-231 and BT-549) in a 35 cm dish, with the growth medium changed every three days. After 14 days of continuous culture, fix the cells with methanol (CH3OH ≥ 99%) for 15 min, followed by staining with 1% crystal violet solution (C8470, Solarbio, Beijing, China) for 10 min. Rinse thoroughly with running water, air dry the plates, and perform statistical analysis. Transwell assay: the MDA-MB-231 and BT-549 cells (2.5 × 10^4^) in serum-free medium were seeded into the upper chambers of transwell inserts (8 μm pore size; Corning, NY, USA). After passing through the membrane, cells were fixed with methanol (CH3OH ≥ 99%), stained with 1% crystal violet, and examined using an inverted microscope (Nikon Instruments Inc., Eclipse Ti2, Melville, NY, USA). Wound healing assay: The cells, MDA-MB-231 and BT-549, were introduced into 6-well or 24-well plates and cultured until they formed a monolayer that was 90%–100% confluent. Using a sterile 200 μL pipette tip (or an appropriately sized one), a straight scratch (wound) was made on the cell monolayer. PBS (pH: 7.2–7.4, ×1) was used to gently rinse the wells one to two times, removing any detached cells and debris from the wound area. Immediately after scratching (0 h), and then again at 24 h, images were captured using a Leica DMI40008B microscope (Leica, Wetzlar, Germany). Cell migration rate (%) was calculated using the following equation: Migration rate (%) = [(scratch width at 0 h − scratch width at 24 h)/scratch width at 0 h] × 100% [15,16].

### 2.11 Experiments with Animals

Sixteen female BALB/c nude mice, aged 3–4 weeks, were obtained from Beijing Vital River Laboratory Animal Technology Co., Ltd., (Beijing, China). Under controlled conditions, the mice experienced a 12-h light/dark cycle, temperatures of 22°C–25°C, and humidity of 50%–70%.

MDA-MB-231 cells were seeded (2 × 10^5^ cells per well) in 10 cm dishes and transfected with either 200 nM siRNA targeting MRPL18 or 25 μg of the MRPL18 overexpression plasmid. For the control groups, 200 nM of negative control siRNA or 25 μg of an empty vector was used for transfection. After incubating for 24 h, the cells were harvested. The cells were subsequently injected into the dorsal area under the skin of nude mice. Tumor volume was assessed weekly and calculated using the equation: V = 0.5 × length × width^2^. Following a period of 28 days, the mice were anesthetized and executed by a 1% pentobarbital sodium overdose, and the tumor tissues were harvested for later examination. Approval for all experimental protocols was granted by the Ethics Committee at Central South University (CSU-2024-0193).

### 2.12 Immunofluorescence (IF) Staining

Harvest the tissue and promptly immerse it in 4% paraformaldehyde in PBS (pH: 7.2–7.4, ×1) for 4 h at room temperature or overnight. Following fixation, rinse the tissue 5 times in PBS (pH: 7.2–7.4, ×1) for 4 min per wash. Embed the fixed tissue in a paraffin compound. Sectioning the tissue at a thickness of 3 µm using a microtome. Tissue samples underwent antigen retrieval, were blocked, and incubated overnight at 4°C with the Ki67 primary antibody (27309-AP, Proteintech, China; 1:50). Afterward, the cells were treated with a fluorescent secondary antibody (AS007, ABclonal, Wuhan, China; 1:100) for 45 min, and then stained with 4^′^,6-Diamidino-2-Phenylindole (DAPI) solution (10 µg/mL, C0065, Solarbio) for nuclear staining. Fluorescence imaging was performed using a Zeiss microscope (Axio Imager 2, Jena, Germany).

### 2.13 TUNEL Staining

Tissue cells were subjected to TUNEL staining using a TUNEL staining kit (C1086, Beyotime, Shanghai, China). The sections underwent treatment with 20 μg/mL of DNase-free Proteinase K (Beyotime, ST532, 20 mg/mL) for 15 min, followed by three 5-min washes with PBS (pH: 7.2–7.4, ×1). For 60 min, the sections were treated with 50 μL of TUNEL reaction solution at 37°C in the absence of light. Post-incubation, the sections underwent three 5-min rinses with PBS (pH: 7.2–7.4, ×1).

### 2.14 Flow Cytometry Assay

Cells (MDA-MB-231 and BT-549) apoptosis was assessed using an Annexin V-FITC/propidium iodide double staining kit (Vazyme, KGA106, Nanjing, China). After staining with Annexin V-FITC (1–5 µg/mL) and propidium iodide (1–10 µg/mL), the apoptotic rate was measured using a flow cytometer (Cytek Biosciences, Fremont, CA, USA) as previously described [17].

### 2.15 Immune Analysis

Version 4.4.2 of R was used for immune analysis. Using the ssGSEA approach, the infiltration levels of 24 immune cell types were measured in breast cancer samples. Using the ‘ggplot2’ (version 3.4.2) and ‘pheatmap’ (version 1.0.12) packages, the link between MRPL18 expression and immune checkpoint genes was analyzed.

### 2.16 Statistical Analysis

Statistical analyses were performed using GraphPad Prism 9.3 (GraphPad Software, Inc., San Diego, CA, USA). A Student’s *t*-test determined group significance, and Spearman’s correlation coefficient assessed correlations. Results are expressed as mean ± SD, with significance indicated by *p < 0.05, **p < 0.01, and ***p < 0.001. All experiments were repeated three times.

## 3 Results

### 3.1 Bulk and Single-Cell Expression of MRPL18

**Figure 1:**
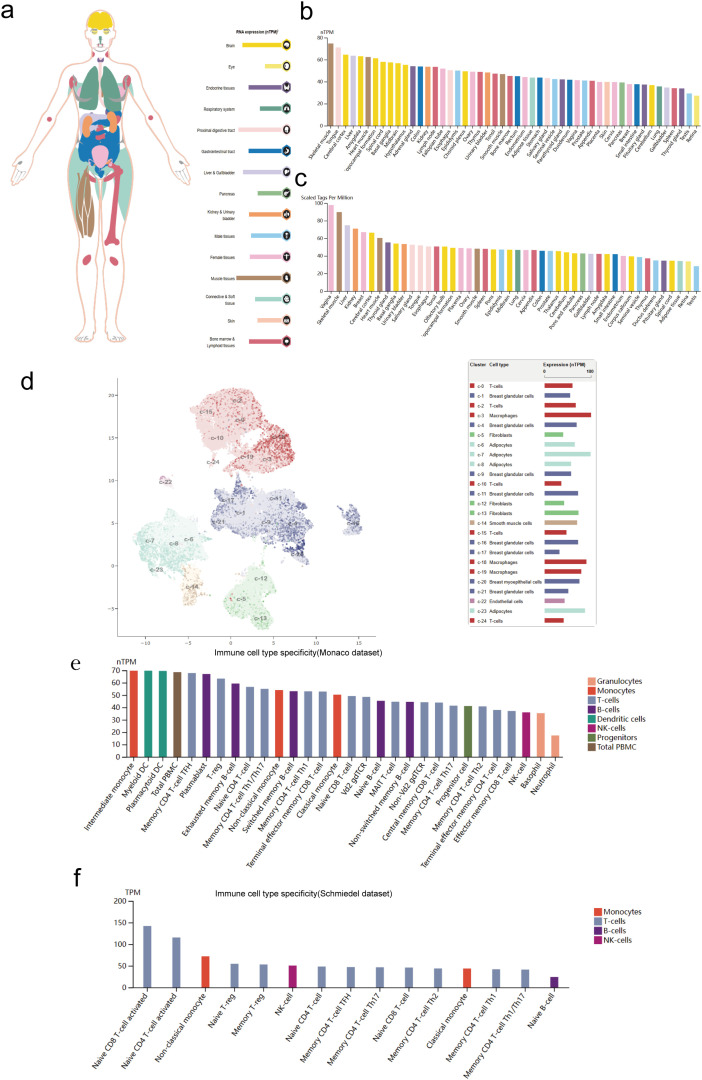
MRPL18 expression level. (**a**,**b**) RNA expression in the HPA database and ConsensusPathDB database; (**c**) The expression of MRPL18 protein, as indicated by the FANTOM5 dataset; (**d**) The single-cell level in breast tissue; (**e**,**f**) The Monaco and Schmiedel datasets

Firstly, the results revealed that MRPL18 was expressed in many human tissues and organs, including muscle tissues, the brain, and the proximal digestive tract (Fig. 1a). The RNA expression of MRPL18 was validated using the ConsensusPathDB. According to the ConsensusPathDB dataset, MRPL18 expression levels were found to be highest in skeletal muscle, the tongue, and the cerebral cortex (Fig. 1b). In FANTOM5 dataset, the findings indicated that the higher expression of MRPL18 were observed in the vagina, skeletal muscle, and liver among all the tissues and organs assessed (Fig. 1c).

Subsequently, the scRNAseq data derived from breast tissues were examined (Fig. 1d). Immune cell type characteristics in hematopoietic tissues were verified using the Monaco and Schmiedel datasets. The Monaco dataset reveals that intermediate monocytes and myeloid dendritic cells have notably increased MRPL18 expression, as shown in Fig. 1e. The schmiedel dataset, as depicted in Fig. 1f, shows that naive CD8 T-cells, naive CD4 T-cells, and non-classical monocytes had the highest MRPL18 expression levels among hematopoietic immune cells.

### 3.2 Significant Overexpression of MRPL18 in Breast Cancer Tissues

**Figure 2:**
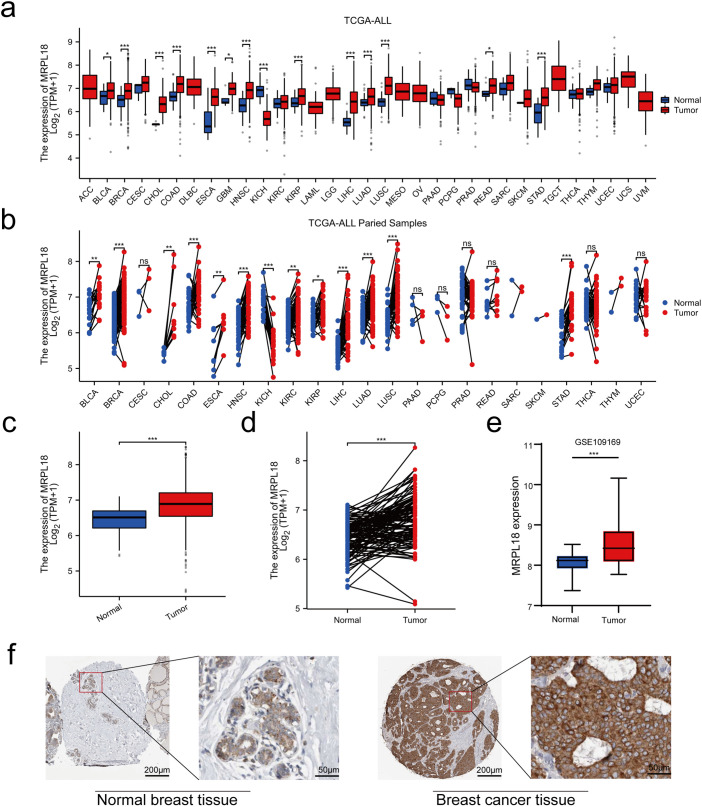

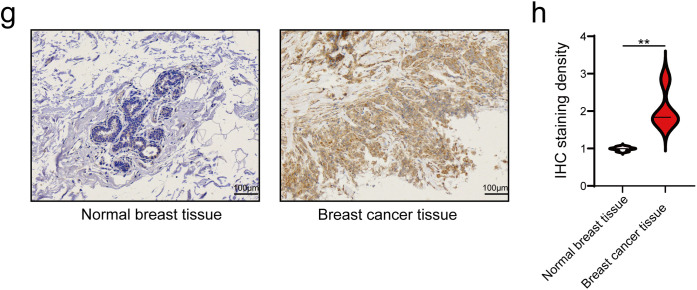
Expression levels and diagnostic significance of MRPL18. (**a**,**b**) MRPL18 expression profiles in various cancers and their corresponding healthy tissue counterparts. (**c**) Comparison of MRPL18 expression in cancerous tissues vs. non-matched healthy tissues. (**d**) In paired samples. (**e**) The GSE109169 dataset. (**f**) IHC photomicrographs of MRPL18 in breast tissue and breast cancer from the HPA database. (**g**,**h**) Example IHC micrographs showing MRPL18 in collected breast and breast cancer clinical samples. *p < 0.05; **p < 0.01; ***p < 0.001; ns, *p* > 0.05, no significant

The disparity in MRPL18 expression between cancerous and healthy tissues is shown in Fig. 2a,b. In addition, by examining data from 1087 patients in TCGA (Table 1), MRPL18 expression was much less in healthy tissues when compared to breast cancer tissues (*p* < 0.001) (Fig. 2c). Besides, MRPL18 was notably elevated in tumor tissue compared to matched normal breast tissue in 112 pairs of samples (Fig. 2d). To strengthen our conclusions, we referenced the GEO database dataset GSE109169, which provided additional support for our main findings (Fig. 2e). To confirm the protein level of MRPL18 in breast cancer using the HPA database, followed by validation of protein level of MRPL18 through immunohistochemistry experiments. The results showed a notable rise in MRPL18 levels in breast cancer tissue (Fig. 2f–h). In conclusion, our study provides strong evidence that MRPL18 is significantly overexpressed in breast cancer tissues.

**Table 1:** Original 1087 breast cancer cases were downloaded from TCGA database

Characteristics	Low expression of MRPL18	High expression of MRPL18	*p*-value
*n*	543	544	
Pathologic T stage, *n* (%)			0.021
T1	160 (14.8%)	118 (10.9%)	
T2	293 (27.0%)	338 (31.2%)	
T3	72 (6.6%)	68 (6.3%)	
T4	17 (1.6%)	18 (1.7%)	
Pathologic N stage, *n* (%)			0.963
N0	258 (24.2%)	258 (24.2%)	
N1	183 (17.1%)	176 (16.5%)	
N2	56 (5.2%)	60 (5.6%)	
N3	38 (3.6%)	39 (3.7%)	
Pathologic M stage, *n* (%)			0.655
M0	452 (48.9%)	453 (49%)	
M1	11 (1.2%)	9 (1.0%)	
Pathologic stage *n* (%)			0.865
Stage I	95 (8.9%)	87 (8.2%)	
Stage II	308 (29.0%)	311 (29.3%)	
Stage III	119 (11.2%)	125 (11.8%)	
Stage IV	10 (0.9%)	8 (0.8%)	
Race, *n* (%)			<0.001
Asian	23 (2.3%)	37 (3.7%)	
Black or African American	71 (7.1%)	111 (11.1%)	
White	405 (40.6%)	350 (35.1%)	
Age, *n* (%)			0.645
≤60	305 (28.1%)	298 (27.4%)	
>60	238 (21.9%)	246 (22.6%)	
Histological type, *n* (%)			0.001
Infiltrating Ductal Carcinoma	360 (36.7%)	416 (42.4%)	
Infiltrating Lobular Carcinoma	121 (12.3%)	84 (8.6%)	
PR status, *n* (%)			0.271
Negative	164 (15.9%)	178 (17.2%)	
Positive	357 (34.5%)	335 (32.4%)	
ER status, *n* (%)			0.333
Negative	114 (11.0%)	126 (12.2%)	
Positive	407 (39.2%)	390 (37.6%)	
HER2 status, *n* (%)			0.609
Negative	284 (39.6%)	276 (38.5%)	
Positive	76 (10.6%)	81 (11.3%)	
PAM50, *n* (%)			<0.001
LumA	307 (29.3%)	257 (24.5%)	
LumB	80 (7.6%)	126 (12%)	
Her2	42 (4%)	40 (3.8%)	
Basal	82 (7.8%)	113 (10.8%)	
Menopause status, *n* (%)			0.217
Pre	127 (13%)	103 (10.6%)	
Peri	20 (2.0%)	20 (2.0%)	
Post	343 (35.1%)	363 (37.2%)	
Anatomic neoplasm subdivisions, *n* (%)			0.413
Left	276 (25.4%)	290 (26.7%)	
Right	267 (24.6%)	254 (23.4%)	

### 3.3 Link between MRPL18 Expression and Clinical and Pathological Features in Breast Cancer

**Figure 3:**
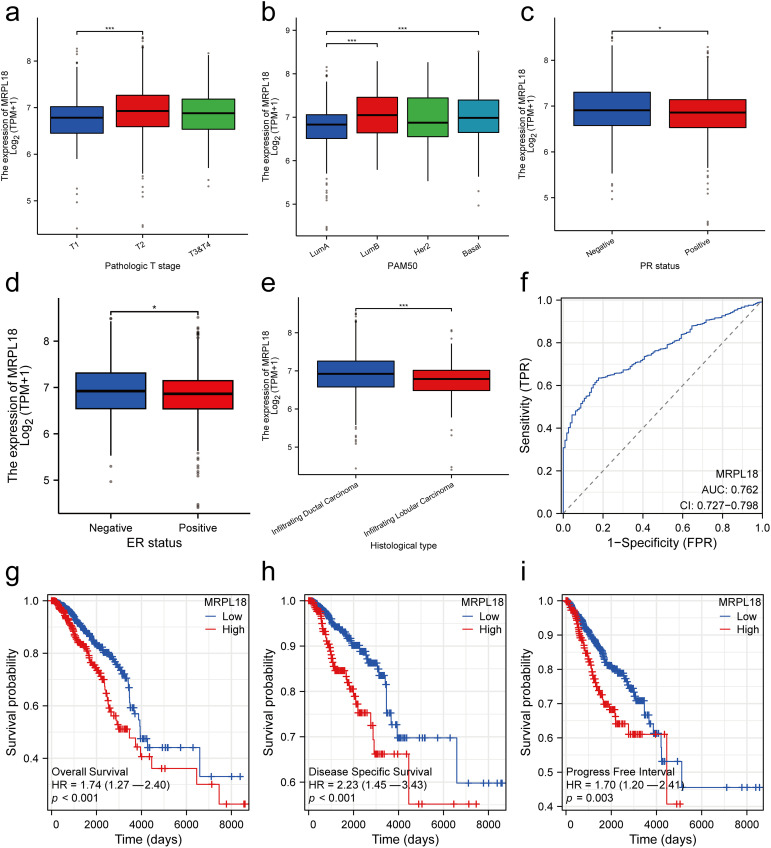

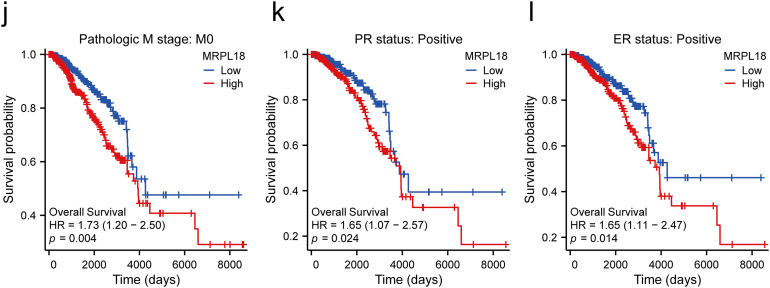
Clinical significance of MRPL18 in breast cancer. The differential expression of MRPL18 was analyzed across various clinical parameters, including (**a**) T stage; (**b**) PMA 50; (**c**) PR status; (**d**) ER status; and (**e**) histological type; (**f**) The AUC value of 0.762; (**g**) OS; (**h**) DSS; (**i**) PFI; (**j**–**l**) in different breast cancer subtypes. *p < 0.05, ***p < 0.001

Subsequently, the clinical results showed that MRPL18 was significantly correlated with T stage, PMA 50 subtype, PR status, ER status, and histological type (all *p* < 0.05), as illustrated in Fig. 3a–e. Notably, MRPL18 expression demonstrated a strong ability to distinguish between cancerous and normal tissues, with an AUC value of 0.762 (Fig. 3f). Furthermore, Kaplan-Meier survival curves indicated that patients with high MRPL18 expression had significantly shorter OS, disease-specific survival (DSS), and progression-free interval (PFI) compared to those with low MRPL18 expression (*p* < 0.05) (Fig. 3g–i). Additionally, higher MRPL18 expression indicates a shorter OS in different subgroups of breast cancer (Fig. 3j–l).

### 3.4 Prognostic Value of MRPL18 in Breast Cancer

**Figure 4:**
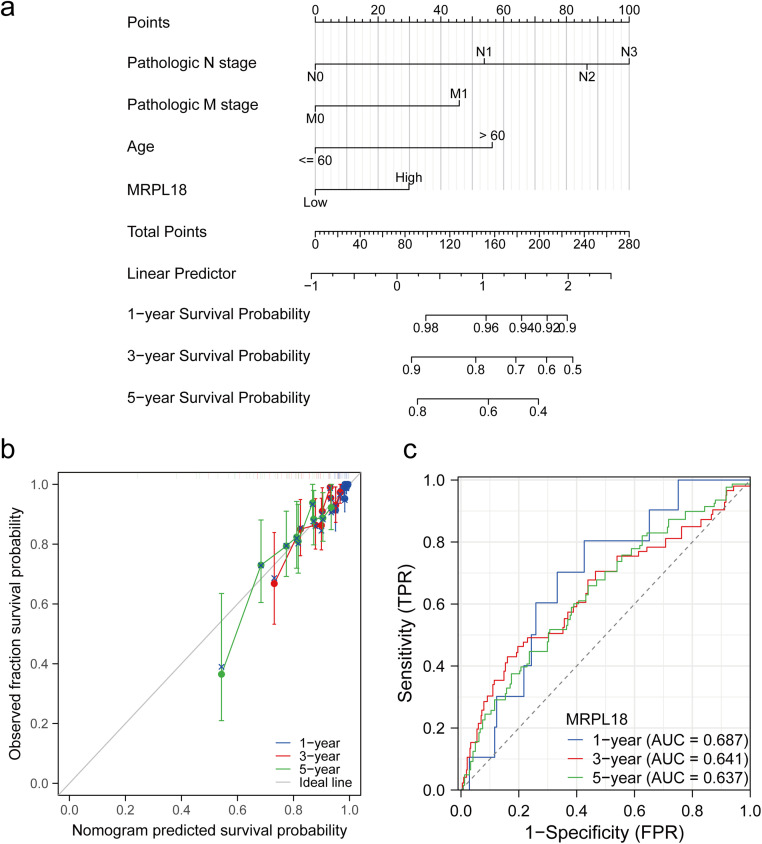
Predictive ability of MRPL18 for breast cancer. (**a**) Nomogram; (**b**) Calibration plots were used to validate the nomogram model; (**c**) Time-dependent ROC analysis

The independent predictive capability of MRPL18 expression was evaluated using univariate and multivariate cox regression models. Analysis indicated that MRPL18, along with N stage, M stage, and age, were significant factors in predicting overall survival in breast cancer (all *p* < 0.05) (Table 2). Subsequently, a nomogram incorporating MRPL18, N stage, M stage, and age was developed. Specifically, nomograms predicting 1-, 3-, and 5-year OS were generated, as shown in Fig. 4a. As the scores for the four prognostic factors increased, patient prognosis progressively worsened. Furthermore, the calibration curve, as shown in Fig. 4b, confirmed the nomogram’s precise prediction of OS. Time-dependent ROC curves confirmed the prognostic significance of MRPL18 in forecasting survival rates (Fig. 4c).

**Table 2:** Univariate and multivariate Cox regression models

Characteristics	total (N)	Univariate analysis		Multivariate analysis	
		Hazard ratio (95% CI)	*p*-value	Hazard ratio (95% CI)	*p*-value
Pathologic T stage	1083				
T1	277	Reference		Reference	
T2	631	1.336 (0.890–2.006)	0.162	1.218 (0.681–2.179)	0.507
T3	140	1.551 (0.921–2.612)	0.099	1.568 (0.722–3.405)	0.255
T4	35	3.759 (1.959–7.213)	<0.001	0.985 (0.327–2.971)	0.979
Pathologic N stage	1067				
N0	516	Reference		Reference	
N1	358	1.947 (1.322–2.865)	<0.001	1.710 (0.992–2.949)	0.054
N2	116	2.522 (1.484–4.287)	<0.001	3.904 (1.969–7.741)	<0.001
N3	77	4.191 (2.318–7.580)	<0.001	6.582 (2.803–15.454)	<0.001
Pathologic M stage	925				
M0	905	Reference		Reference	
M1	20	4.266 (2.474–7.354)	<0.001	3.189 (1.290–7.884)	0.012
Age	1086				
≤60	603	Reference		Reference	
>60	483	2.024 (1.468–2.790)	<0.001	3.075 (1.686–5.608)	<0.001
Histological type	981				
Infiltrating Ductal Carcinoma	776	Reference			
Infiltrating Lobular Carcinoma	205	0.830 (0.528–1.304)	0.420		
PR status	1033				
Negative	342	Reference		Reference	
Positive	691	0.729 (0.521–1.019)	0.065	0.780 (0.366–1.664)	0.520
ER status	1036				
Negative	240	Reference		Reference	
Positive	796	0.709 (0.493–1.019)	0.063	0.528 (0.221–1.262)	0.151
PAM50	1046				
LumA	563	Reference		Reference	
LumB	206	1.664 (1.089–2.542)	0.019	1.163 (0.643–2.101)	0.618
Her2	82	2.270 (1.330–3.875)	0.003	0.889 (0.303–2.608)	0.831
Basal	195	1.290 (0.836–1.989)	0.250	1.009 (0.388–2.624)	0.986
Menopause status	935				
Pre	230	Reference		Reference	
Post	705	2.153 (1.295–3.581)	0.003	1.095 (0.526–2.279)	0.809
Anatomic neoplasm subdivisions	1086				
Left	566	Reference			
Right	520	0.770 (0.557–1.063)	0.112		
MRPL18	1086				
Low	543	Reference		Reference	
High	543	1.601 (1.154–2.222)	0.005	1.762 (1.117–2.779)	0.015

### 3.5 Functional Analysis

**Figure 5:**
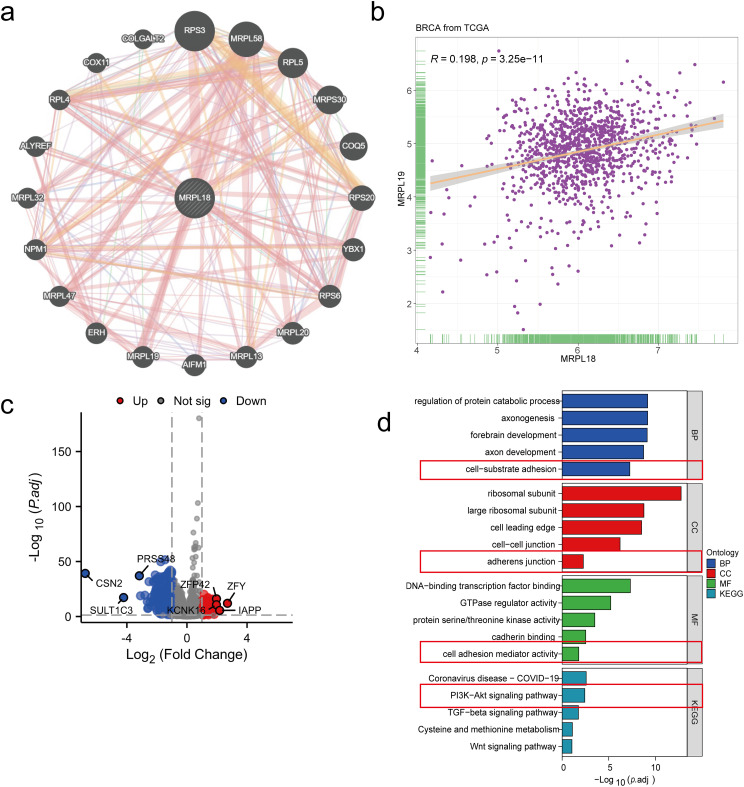

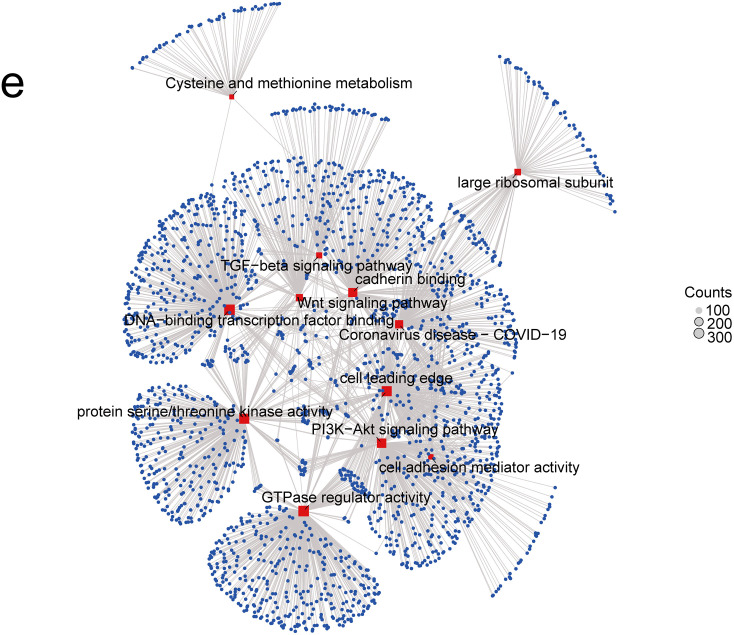
Functional analysis of MRPL18. (**a**) PPI network of MRPL18; (**b**) Correlation analysis; (**c**) Volcano plots illustrating differential gene expression among various MRPL18 expression subgroups; (**d**) GO and KEGG analysis results; (**e**) KEGG network diagram

A PPI network was initially constructed to identify 20 hub genes related to MRPL18, including MRPL19, as showed in Fig. 5a. A positive correlation between MRPL18 and MRPL19 was found in the analysis (R = 0.198, *p* < 0.001) (Fig. 5b). Differential analysis was performed to generate volcano plots comparing samples with high and low MRPL18 expression, as shown in Fig. 5c. The GO and KEGG revealed that MRPL18 is associated with cell-substrate adhesion, adherens junctions, cell adhesion mediator activity, the PI3K-AKT signaling pathway, proliferation, metastasis, and other functions, as depicted in Fig. 5d,e.

### 3.6 MRPL18 Promotes the Proliferation and Migration of Breast Cancer In Vitro

**Figure 6:**
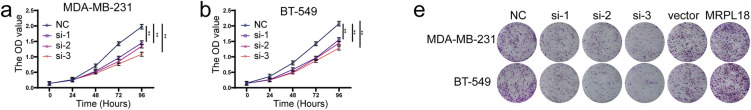

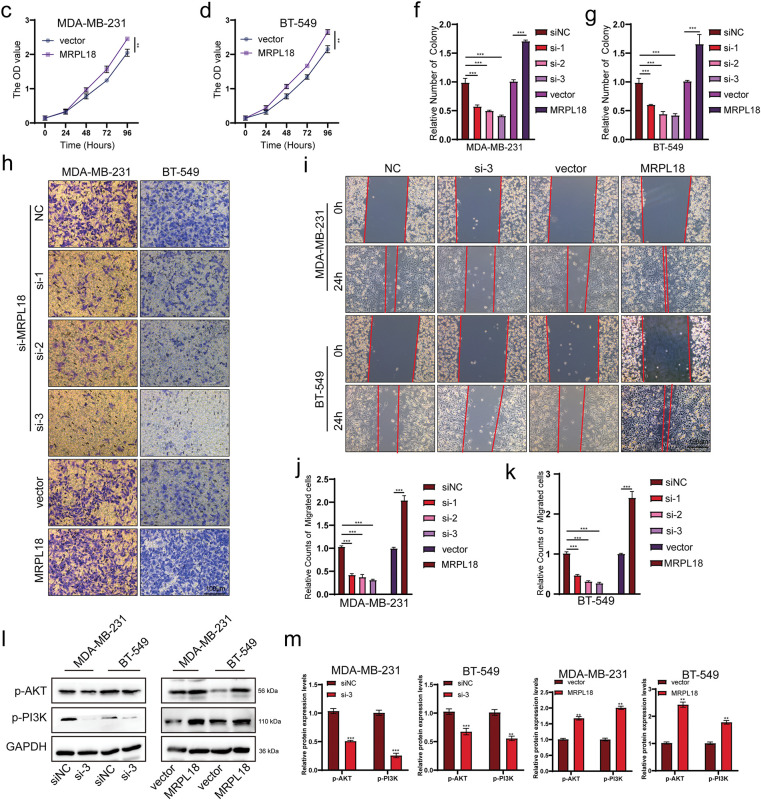
MRPL18 promotes proliferation and migration in breast cancer cells. (**a**–**g**) CCK-8 assay and colony formation assay. (**h**) Transwell assays (scale bar = 100 µm). (**i**) Wound healing assays (scale bar = 100 µm). (**j**,**k**) Quantitative analysis of counts of migrated cells. (**l**,**m**) Western blotting assays. **p < 0.01, ***p < 0.001

To investigate the effects of MRPL18 on cellular functions, a variety of *in vitro* experiments were carried out to assess its influence on cell proliferation, migration, and apoptosis. After transfecting cells with the MRPL18 overexpression plasmid, the results showed a significant upregulation of MRPL18 expression. Conversely, MRPL18 expression was markedly reduced after being transfected with three specific siRNA sequences (Fig. S1). Moreover, CCK-8 and colony assays showed that overexpression of MRPL18 was found to promote cell proliferation and colony formation ability, while MRPL18 knockdown significantly decreased these abilities (Fig. 6a–g).

Traswell and wound healing assays showed that MRPL18 promotes cell migration ability, while knockdown of MRPL18 was found to suppress the migratory ability of breast cancer cells (Figs. 6h–k and S2). Flow cytometry results showed that si-MRPL18 promoted cell apoptosis, while overexpression of MRPL18 inhibited apoptosis (Fig. S3). Additionally, the western blotting results showed that reducing MRPL18 levels significantly inhibited the PI3K-AKT pathway, while increasing MRPL18 levels enhanced the pathway’s activation (Fig. 6l,m).

### 3.7 MRPL18 Promotes Tumor Growth in Nude Mice

**Figure 7:**
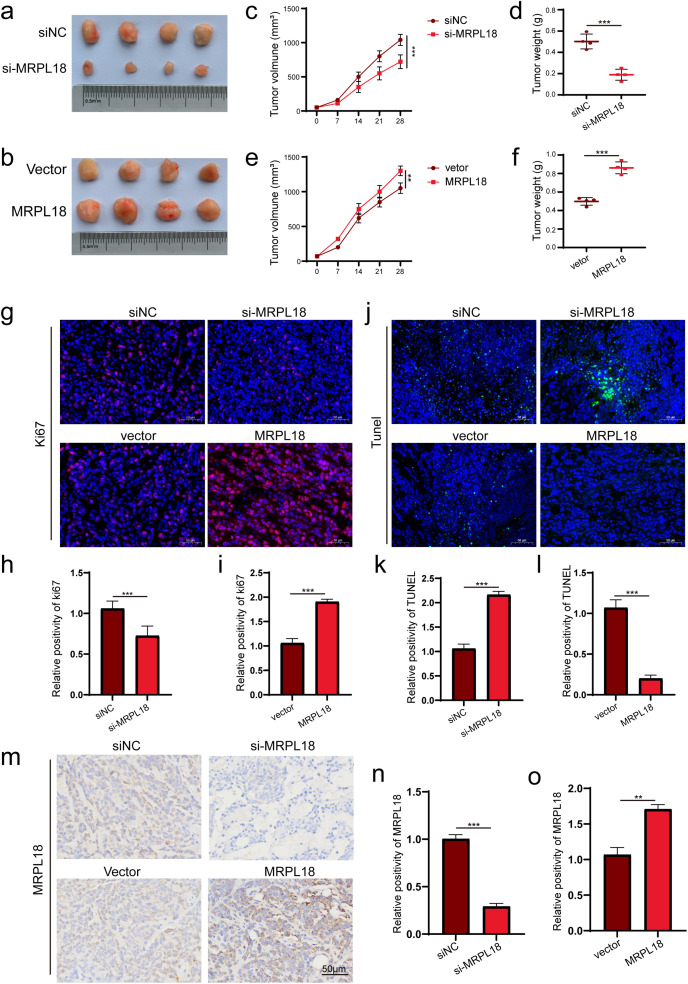
The effect of MRPL18 on tumor growth *in vivo*. (**a**,**b**). Photographs of tumor specimens. (**c**–**f**). Assessment of tumor volume and weight regarding MRPL18 expression. (**g**–**i**). Immunofluorescence was employed to evaluate the expression of Ki-67. (**j**–**l**). TUNEL assay. (**m**–**o**). IHC assays. **p < 0.01, ***p < 0.001

The xenograft tumor model using nude mice to explore MRPL18’s impact on *in vivo* tumor growth. (Fig. 7a,b). The results showed that subcutaneous tumors had notably reduced volume and weight in the si-MRPL18 group compared to the negative control group, while overexpression of MRPL18 tumor growth (Fig. 7c–f). Immunofluorescence experiments revealed a significant suppression of Ki67 expression in the si-MRPL18 compared to the negative control group, while Ki67 expression was significantly upregulated in the MRPL18-OE group compared to the vector control group (Fig. 7g–i). TUNEL experiments revealed that MRPL18 knockdown promotes cell apoptosis, while overexpression of MRPL18 suppresses cell apoptosis (Fig. 7j–l). Additionally, The MRPL18 protein level significantly decreased in the si-MRPL18 group compared to the negative control group, while it significantly increased in the MRPL18-OE group (Fig. 7m–o).

### 3.8 Immune Response

**Figure 8:**
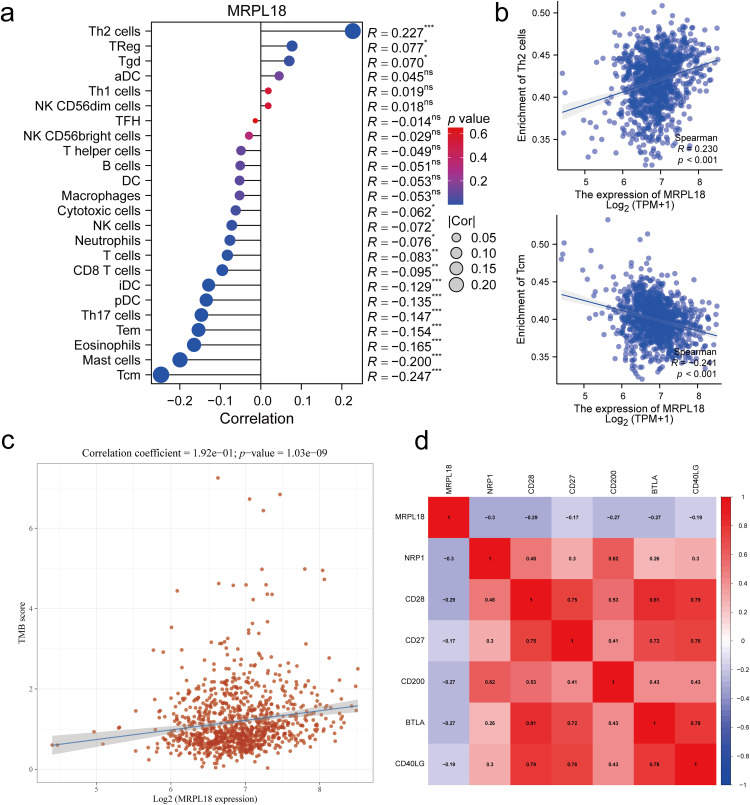
Correlation analysis of immune response. (**a**) 24 immune cell types; (**b**) Th2 cells and Tcm cells (Tcm); (**c**) Tumor mutation burden; (**d**) The key immune checkpoint genes. *p < 0.05, **p <0.01, ***p <0.001, ns, *p* > 0.05, no significant

Single-sample GSEA was conducted to assess how MRPL18 expression influences immune infiltration within the tumor microenvironment. Spearman’s correlation analysis showed a positive relationship between MRPL18 expression and the infiltration levels of different immune cells, including Th2 cells, but it also had a negative correlation with the infiltration levels of certain immune cells, like central memory T cells (Tcm) cells (*p* < 0.05) (Fig. 8a,b). Furthermore, MRPL18 expression was positively correlated with tumor mutation burden (*p* < 0.05) (Fig. 8c). Finally, we identified a negative correlation with genes such as NRP1, CD28, and CD200 (*p* < 0.05) (Fig. 8d). MRPL18 may promote an immunosuppressive tumor microenvironment, potentially reducing the efficacy of immunotherapy.

## 4 Discussion

Breast cancer is the most frequently diagnosed cancer in 159 nations and the top cause of cancer-related deaths in 110 countries [18,19]. Individuals with early-stage breast cancer are commonly treated with surgical procedures, while chemotherapy is typically administered to those with advanced-stage cancer, known for its low 5-year survival rate [20]. Enhancing the effectiveness of chemotherapy, improving targeted therapies, and optimizing prognostic management are crucial for improving outcomes in patients. New targets could lead to improved methods for diagnosing and treating breast cancer.

Changes in mitochondrial energy metabolism and apoptosis, which are key cellular functions associated with cancer, are disrupted by genomic instability [21]. The translation process in mitochondria depends significantly on mitochondrial ribosomal proteins (MRPs) [22]. Previous studies have shown that more than 40 MRPs are overexpressed in breast cancer, where they act as enhancers of cellular viability [23]. For example, MRPL33 and its splicing regulator play a crucial role in colorectal cancer cells by promoting their growth and proliferation while inhibiting apoptosis [24]. Likewise, MRPL35 is abundantly expressed in colorectal cancer, and its suppression leads to hindered cell proliferation and ultimately cell death [25]. MRPL18’s function and mechanism in tumors remain largely unexamined.

This study comprehensively explored the role of MRPL18 in breast cancer by analyzing its expression data from multiple databases. MRPL18 is highly expressed in breast cancer and is a risk factor. To further investigate its functional role, a PPI network was constructed, identifying 20 hub genes potentially associated with MRPL18, RPS3 [26], MRPL58, RPL5, MRPS30, COQ5, RPS20, RPS6, MRPL20, MRPL19, YBX1 [27–29], AIFM1, ERH, NPM1, MRPL13 [30], ALYREF, MRPL32, RPL4, COX11, COLGALT2 [31] and and MRPL47. Several of these hub genes have been shown to exhibit pro-tumorigenic effects and are positively correlated with MRPL18 expression. For instance, Increased MRPL19 expression in lung adenocarcinoma was connected to lymph node metastasis [32]. Furthermore, functional enrichment analysis revealed that MRPL18 is involved in pathways critical to tumor survival and metastasis, including cell-substrate adhesion [33], adherens junction [34], cell adhesion mediator activity [35], PI3K-AKT pathway [36].

Following CCK-8 and cloning experiments revealed that MRPL18 knockdown notably hindered cell proliferation, while its abnormal expression encouraged breast cancer cell growth. Additionally, transwell migration assays confirmed that MRPL18 enhances the migration ability of breast cancer cells. The PI3K signaling pathway, known to be aberrantly activated in various diseases, particularly cancer, has been shown to promote tumor proliferation and metastasis [37,38]. The research revealed that MRPL18 plays a role in activating the PI3K pathway, further implicating it in oncogenic processes. Consistent with *in vitro* findings, it showed that overexpression of MRPL18 accelerated tumor growth and increased expression of Ki67 in tumor tissues. Taken together, these bioinformatic and experimental results demonstrate that MRPL18 promotes breast cancer cell proliferation and migration, supporting its function as a potential oncogene. According to the immune surveillance model, immune cells possess the ability to recognize and eliminate tumor and neoplastic cells [39]. However, tumors can evade immune-mediated destruction through mechanisms classified into three phases: elimination, equilibrium, and escape. Once immune evasion occurs, tumor progression typically accelerates. Cancer immunotherapies, such as monoclonal antibodies and tumor vaccines, aim to enhance the host’s anti-tumor immune response, thereby facilitating tumor eradication [40]. MRPL18 expression was linked to the infiltration of several immune cells, including Th2 cells, in this study. Notably, targeting Th2-mediated immunity has been shown to enhance the efficacy of immunotherapy in breast cancer [41]. Furthermore, recent studies have highlighted a strong association between TMB and clinical outcomes in breast cancer patients [42]. Aligned with this observation, our analysis identified a strong positive correlation between MRPL18 expression and TMB levels, implying a possible influence of MRPL18 on the tumor’s immune context. Molecules or signaling pathways known as immune checkpoints regulate the activation of the immune system, playing a key role in preserving immune stability and preventing autoimmunity [43]. Interestingly, MRPL18 expression was negatively correlated with several key immune checkpoint molecules, indicating its potential involvement in immune regulation and suggesting implications for immunotherapeutic response. These findings provide a foundation for exploring immunotherapy strategies tailored to MRPL18 expression profiles in breast cancer. Additionally, studies have demonstrated that anti-TGF-β/PD-L1 synergize with radiotherapy to enhance anti-tumor immunity while reducing I-induced pulmonary fibrosis [44]. Additionally, Li et al. reported that amplification of the MYC gene increases the sensitivity of triple-negative breast cancer to CHK1 inhibitors [45]. Consequently, future development of inhibitors aimed at the MRPL18 protein may increase the effectiveness of radiotherapy and chemotherapy in breast cancer treatment.

**Figure 9:**
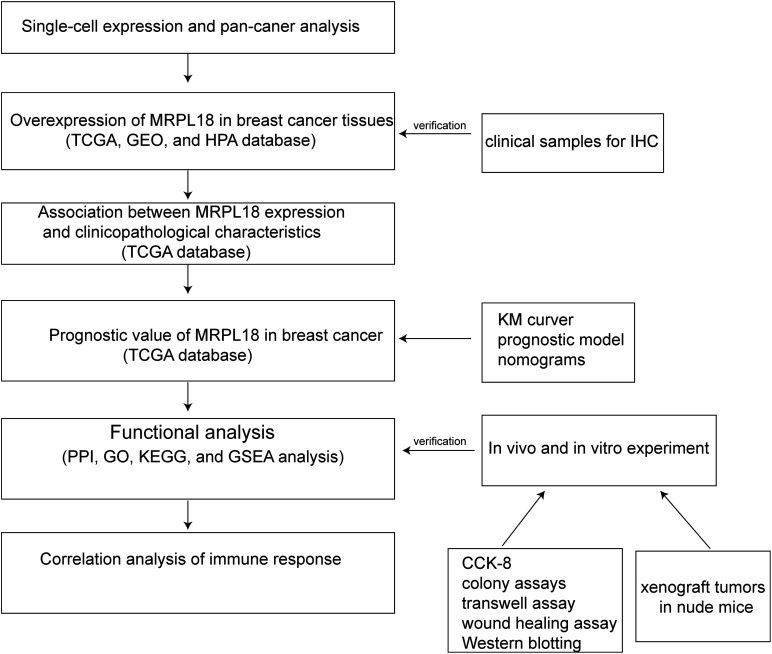
Flow chart of the analysis program used in this study

Even though an initial study of the molecular mechanisms has been conducted, acknowledging certain limitations is essential. The complete understanding of effective methods to manage breast cancer development and progression is still lacking. The molecular data presented in this study serve primarily as preliminary evidence and are not yet sufficient to establish definitive conclusions. This research establishes a foundation for creating new therapeutic approaches, highlighting the need for additional investigation into MRPL18 and its related molecular network. Ultimately, targeting MRPL18 may offer a viable strategy for improving clinical outcomes and advancing the field of breast cancer therapy (Fig. 9). With the rapid advancements in biomaterials and tissue engineering technologies, organoid research and its applications have progressed significantly [46]. In subsequent studies, we might utilize organoids as a model to investigate the regulatory mechanisms of MRPL18 in the occurrence and progression of breast cancer, aiming to provide novel insights for personalized medicine.

## Supplementary Materials

Figure S1 Assessment of MRPL18 knockdown and overexpression efficiency. **(a-b)** Western blot analysis was conducted to evaluate MRPL18 expression in breast cancer cell lines. **(c-d)** Statistical analysis was performed to determine MRPL18 expression levels. All data are presented as mean ± SD, derived from at least three independent experiments. ***p < 0.001.

Figure S2 Statistical analysis of wound healing assays. **(a)** Low MRPL18 expression group. **(b)** High MRPL18 expression group. All data are presented as the mean ± SD, derived from at least three independent experiments. **p < 0.01, ***p < 0.001.

Figure S3 The indicated cells were subjected to Annexin V-APC and PI staining to detect the apoptotic rate by flow cytometry. **(a)** knockdown of MRPL18 in MDA-MB-231 and BT-549. **(b)** overexpression of MRPL18 in MDA-MB-231 and BT-549. **(c-f)** Statistical analysis of cell apoptosis rate. ***p< 0.001.

## Data Availability

The datasets used and/or analyzed during the current study are available from the corresponding author on reasonable request.

## Supplementary Materials

The supplementary material is available online at https://www.techscience.com/doi/10.32604/or.2025.065050/s1.
