# Supplementary figures and images for: MRPL18 Promotes Breast Cancer Progression: Connecting Mitochondrial Ribosomal Protein to Immune Response
Source: Oncol Res. 2025 Aug 28;33(9):2549–71. doi: 10.32604/or.2025.065050 (PMC12408865; doi:10.32604/or.2025.065050)

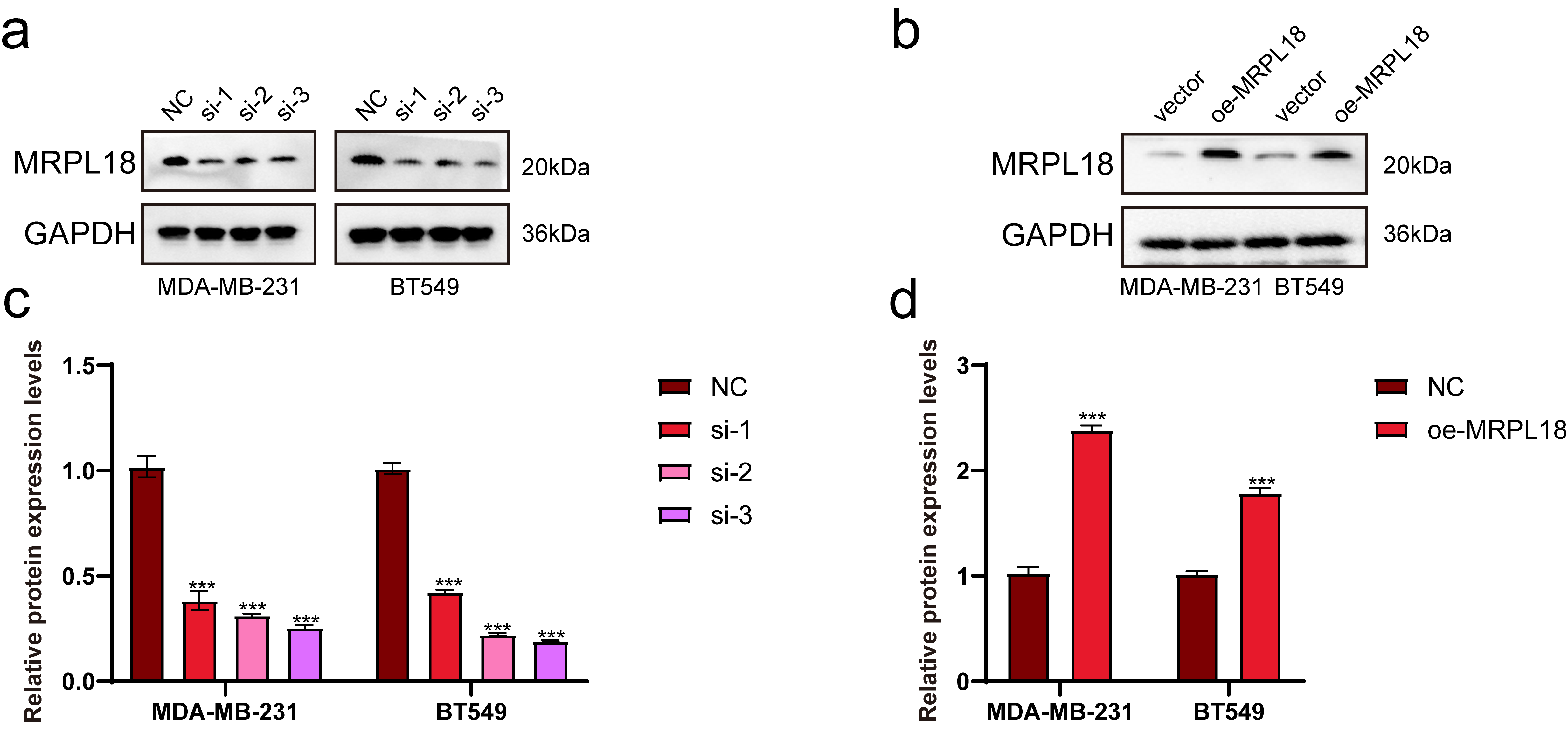

Supplement: Figure S1 [file OncolRes-33-65050-s001.tif]

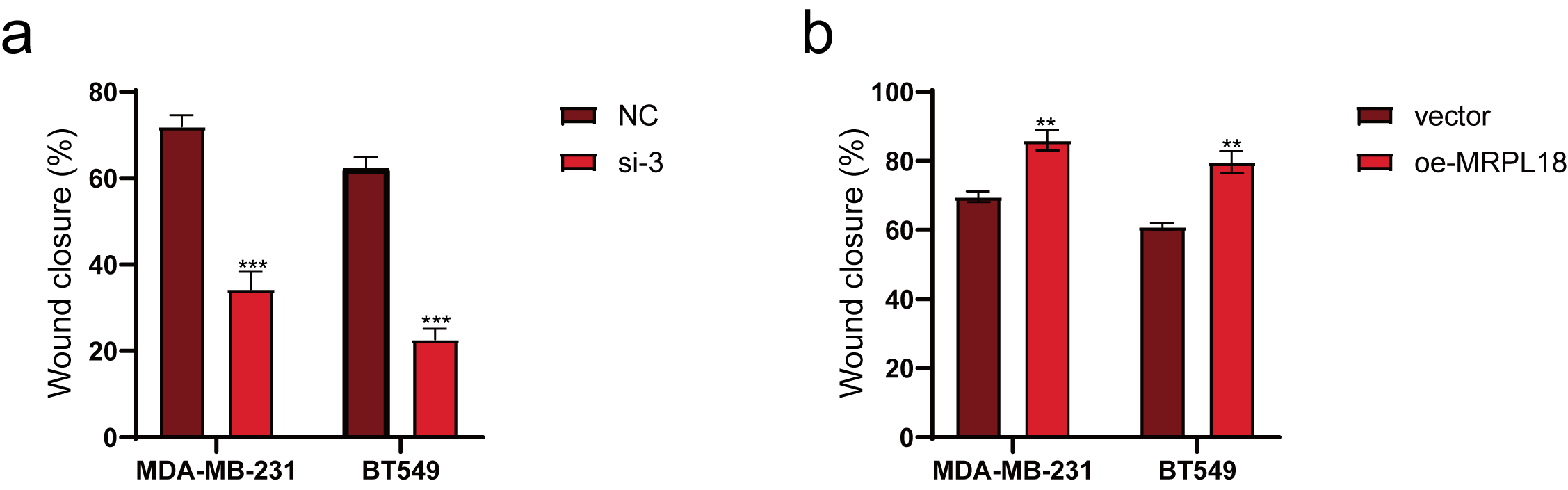

Supplement: Figure S2 [file OncolRes-33-65050-s002.tif]

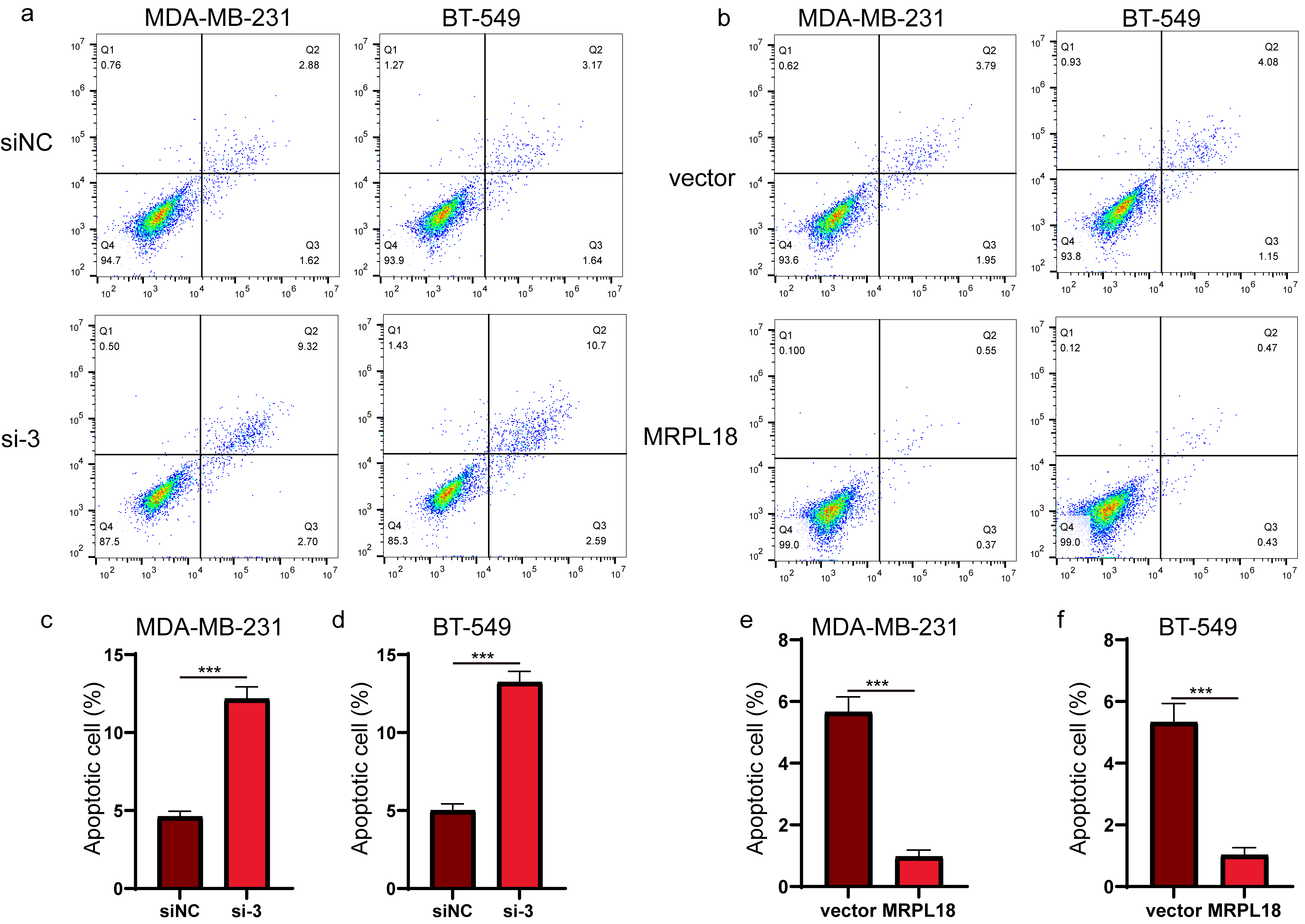

Supplement: Figure S3 [file OncolRes-33-65050-s003.tif]
