# Supplementary material for: MRPL18 Promotes Breast Cancer Progression: Connecting Mitochondrial Ribosomal Protein to Immune Response
Source: Oncol Res. 2025 Aug 28;33(9):2549–71. doi: 10.32604/or.2025.065050 (PMC12408865; doi:10.32604/or.2025.065050)
Supplement: Supplementary file 4 [file OncolRes-33-65050-s004.docx]

Supplemental Files:


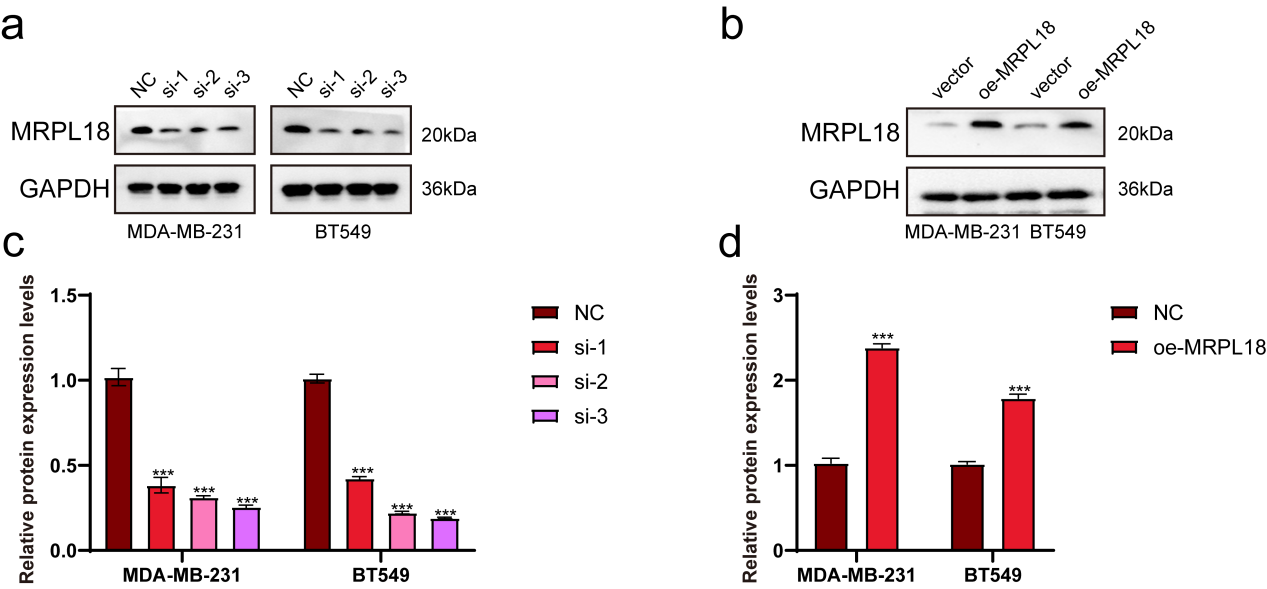


**Figure S1.** Assessment of MRPL18 knockdown and overexpression efficiency. **(a-b)** Western blot analysis was conducted to evaluate MRPL18 expression in breast cancer cell lines. **(c-d)** Statistical analysis was performed to determine MRPL18 expression levels. All data are presented as mean ± SD, derived from at least three independent experiments. ****p* < 0.001.


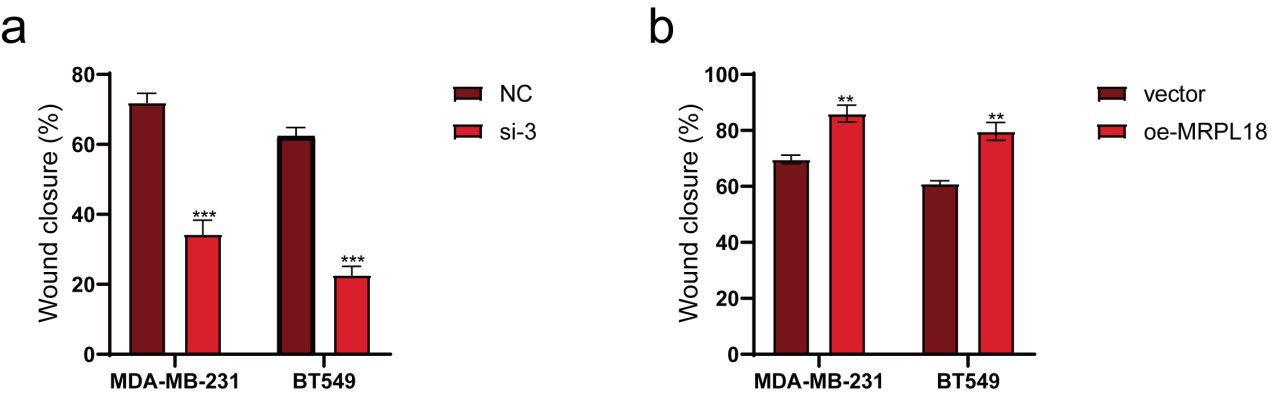


**Figure S2.** Statistical analysis of wound healing assays. **(a)** Low MRPL18 expression group. **(b)** High MRPL18 expression group. All data are presented as the mean ± SD, derived from at least three independent experiments. ***p* < 0.01, ****p* < 0.001.


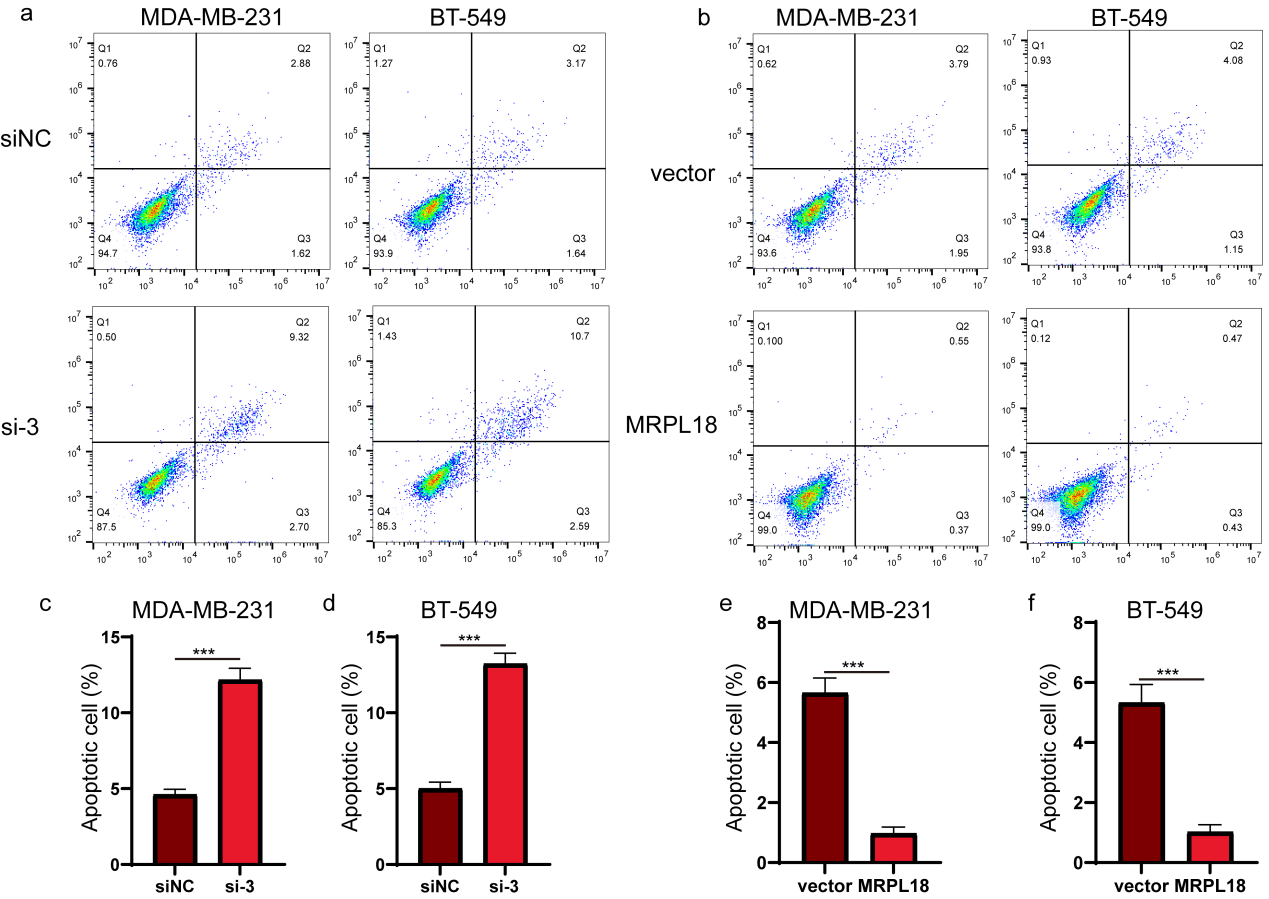


**Figure S3.** The indicated cells were subjected to [Annexin](https://www.sciencedirect.com/topics/biochemistry-genetics-and-molecular-biology/annexin) V-APC and PI staining to detect the apoptotic rate by flow cytometry. **(a)** knockdown of MRPL18 in MDA-MB-231 and BT-549. **(b)** overexpression of MRPL18 in MDA-MB-231 and BT-549. **(c-f)** Statistical analysis of cell apoptosis rate. ****p* < 0.001.

**Supplementary Table 1:** Primer sequence.

| Gene | Forward primer (5′–3′) | Reverse primer (5′–3′) |
| --- | --- | --- |
| MRPL18-qPCR | TGGCACAGGTTGCGAGTTAT | CCCGCCTCTAAGCATCTCTG |
| MRPL18-CDS | AAGCTTATGGCGCTTCGGTCGCGGT | GGATCCTTATTCATAGATTCTCTGAGG |
